# Optimal Design of a Novel Composite Anchorage for Carbon-Fiber-Reinforced Polymer (CFRP) Tendons

**DOI:** 10.3390/polym14102048

**Published:** 2022-05-17

**Authors:** Yamin Sun, Kuihua Mei, Shengjiang Sun, Tao Wang, Xiang Ren

**Affiliations:** 1 School of Architecture & Civil Engineering, and Postdoctoral Research Station on Civil Engineering, Xi’an University of Science & Technology, Xi’an 710054, China; renxiang798@xust.edu.cn; 2 School of Highway, Chang’an University, Xi’an 710064, China; meikuihua@chd.edu.cn (K.M.); sunshengjiang@chd.edu.cn (S.S.); wtbridge@chd.edu.cn (T.W.)

**Keywords:** novel composite anchorage, CFRP tendons, optimal design, experimental, FE methods, parametric study

## Abstract

In this study, we proposed a novel composite anchorage that considers the anchoring performance and dimension simultaneously. The design concept of this composite anchorage was first introduced, followed by comparison with the traditional inner-cone bond-type anchorage and traditional composite anchorage through theoretical and experimental methods. Then, a parametric study was conducted to determine the influence of different parameters on the anchoring performance, and the optimal design parameters were recommended according to the finite element (FE) and test results. Finally, the practicability of the optimal design parameters were validated through experiments on the anchorage with multiple CFRP tendons. Results showed that the novel composite anchorage could improve the anchoring performance compared with the traditional inner-cone bond-type anchorage by promoting increased anchorage efficiency by 60.4% and, with an ideal failure mode of tendon rupture. Moreover, the novel composite anchorage had smaller dimensions and avoided the presence of a vulnerable position at the junction of the mechanical and bond parts compared with the traditional composite anchorage. In addition, a group of optimal design parameters of this composite anchorage with a pre-tightening force of 130 kN, an inclinational differential angle of 0.1°, an inclination angle of 2.9°, and an embedded length of 30 *d*~40 *d* were proposed. The composite anchorage with five CFRP tendons designed with the proposed parameters failed with the rupture of the tendons and exhibited an anchoring efficiency of 1.05. This result showed that the optimal parameters were suitable for this novel composite anchorage to grip multiple tendons. This study can provide an experimental and theoretical basis for designing large-tonnage anchorage for multiple FRP tendons used as hangers or cables in real bridges.

## 1. Introduction

Fiber-reinforced polymer (FRP) has superior properties, such as lightweight, high tensile strength, corrosion resistance, and anti-fatigue performance. FRP can solve the problems of high self-weight and poor durability for bridge structures using traditional steel cable systems [1,2,3]. FRP can largely reduce the self-weight of bridge structures and thus promote spanning ability. In addition, the superior durability of FRP extends the maintenance period and saves the life-cycle maintenance cost of bridge structures [4,5]. Hence, FRP has been regarded as an optimal substitute for traditional steel cable systems in bridge engineering since its inception.

However, the compressive and interlaminar shear strengths of FRP are relatively low. Anchorages used to grip traditional steel tendons or cables can easily bite on the surface of FRP tendons or cables, leading to premature failure of FRP tendons or cables [6,7,8]. Anchorages suitable for FRP tendons or cables are necessary and need to be innovated to popularize the application of FRP in bridge engineering.

Currently, three types of anchorages for FRP tendons or cables have been proposed and studied by researchers, namely mechanical, bond-type, and composite anchorages [9,10]. Traditional mechanical anchorage for steel tendons are improved by placing a soft (aluminum or copper) tube between the spikes and the tendon or cable to prevent the spikes from biting on the surface of the tendon or cable [11]. However, the mechanical anchorage is sensitive to manufacture and assembly errors, where a small error may cause unexpected failure of the FRP tendon [12]. Bond-type anchorages are composed of a steel tube, grout material, and the FRP tendons or cables. For bond-type anchorages, the bond strength between the grout material and FRP tendons or cables determines the entire performance of the anchorage [13]. Researchers have attempted to enhance the bond between the FRP tendon (cable) and the grout material with methods such as using sand-coated FRP tendons [14], scattered-end tendons [15,16], and additive mixed grout material [17,18]. However, the most common enhancement method is promoting the friction force between the FRP tendon (cable) and grout material by enlarging the interfacial contact pressure. To achieve this goal, a straight steel tube has been optimized with constant decreasing inner radii along the longitudinal direction to form an inner cone anchorage [19,20,21]. The inner-cone steel tube can exert a confinement effect on the grout material when it deforms forward under pullout load, thus promoting interfacial contact pressure and load-carrying capacity. For inner cone anchorages, a stress concentration may occur at the loaded end, and the FRP tendon would be cut off before the tendon reaches its ultimate tensile strength [22,23]. To solve this problem, several methods, such as adopting an inner-cone straight tube [24,25,26], curved inner-face tube [27], and grout material with gradient stiffness [28], have been proposed. Furthermore, to take full advantage of mechanical and bond-type anchorages, researchers also have proposed a composite anchorage that combines mechanical and bond-type anchorages in the radial direction [29,30,31,32]. The mechanical part can exert active confinement on the bond part and thus can largely promote interfacial contact pressure between the grout material and FRP tendon. To control the size of the anchorage, the mechanical part of the composite anchorage only grips part of the bond part [29,30,31,32] and would cause a vulnerable position at the junction of the mechanical and bond parts [32].

Although extensive research has been conducted on anchorage innovation, FRP has not yet been widely applied in bridge engineering. The main reason is that an anchorage that can grip FRP tendons successfully with suitable dimensions has not yet been invented. For demonstration bridges with FRP cable or hangers, the dimensions of anchorages for gripping FRP cables or hangers are much larger than those of anchorages for traditional steel cables or hangers. (e.g., the GA 12–20 anchorage for gripping CFRP hangers in Aizhai bridge has a diameter of 27 cm and an embedded length of 73.7 cm [1], whereas the anchorage for gripping traditional steel hangers in this bridge has a diameter of only 19 cm and an embedded length of 48.2 cm). On the one hand, extremely large anchorage dimensions affect its applicability in bridge engineering. On the other hand, for bond-type anchorages, the enhancement effect of anchorage length on the load-carrying capacity is constrained when the anchorage length exceeds a certain value [5,33,34]. In other words, simply enlarging the anchorage dimensions to improve anchorage performance is not always beneficial; on the contrary, it would affect the applicability. Hence, for a wide application of FRP cables or tendons in bridge engineering, an anchorage system that can grip the FRP cables or tendons successfully with applicable dimensions needs to be proposed. For a given anchorage, performance and optimal dimension parameters also need to be studied and determined. However, determining the anchorage performance and optimizing the anchorage design parameters by experiments only are time-consuming and costly processes. The finite element (FE) method is an effective tool to study anchorage properties and has been adopted by many researchers in combination with experimental studies [19,30,35,36,37].

In this study, we proposed a novel composite anchorage with the aim of gripping the CFRP tendon successfully with applicable dimensions. This novel composite anchorage was designed to improve anchoring efficiency with a slightly larger diameter but a much shorter embedded length compared with traditional bond-type anchorages. The novel composite anchorage was designed to have a more uniformly distributed contact pressure among anchorage assemblies and to avoid vulnerable positions at the junction of the mechanical and bond parts compared with traditional composite anchorages. The main purpose of this paper is to propose optimal design parameters of this novel composite anchorage. The structure of this paper is as follows: First, the innovativeness of the composite anchorage is introduced and analyzed theoretically. Then, the advantage of this anchorage is proved through tests and FE methods. Third, the influence of design parameters on the anchorage performance is studied through FE analysis, and the optimal design parameters are suggested. Finally, an optimal anchorage with five CFRP tendons is tested to preliminarily verify its effectiveness in anchoring small-tonnage multiple FRP tendons. This study can provide a basis for designing anchorages for multiple FRP tendons as hangers or cables used in real bridges.

## 2. Design Concept of This Novel Composite Anchorage

**Figure 1:**
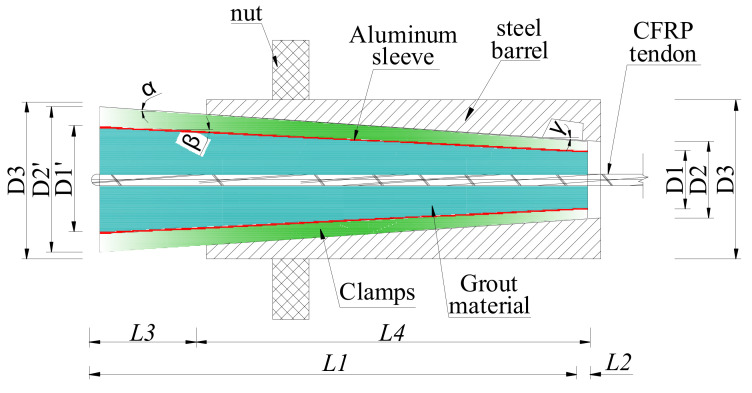
Scheme of the novel composite anchorage.

The novel composite anchorage is composed of five parts, that is, the outer steel barrel, steel clamps, the aluminum sleeve, the grout material, and the CFRP tendon. Figure 1 shows the scheme of this novel composite anchorage. The inner-cone anchorage exhibits a better anchorage performance in reducing slip and promoting anchoring efficiency than a straight anchorage [19,20,21]. Hence, the bond part in this novel composite anchorage was designed as the inner-cone type. An angle differential (α−γ) of 0.1° was set between the outer surface of the clamp and the inner surface of the barrel to address the problem of stress concentration at the loaded end for the inner-cone anchorage. However, the inclination angles of the bond part and the inner surface of the clamp were the same. The outer surface of the clamp and the inner surface of the barrel could not fit each other, and a slight gap existed because of the angle differential, thus preventing the clamps from biting the bond part severely at the loaded end.

In addition, the mechanical part of this novel composite anchorage was designed by setting the thickness of the clamp as almost constant (less than 1 cm) by adjusting to the inner-cone bond part. Before the anchorage was subjected to pullout load, a pre-tightening force was designed to apply to the end of the clamps so that an active confinement effect was exerted to the bond part. Thus, contact pressure between the CFRP tendon and the grout was promoted, and the anchoring performance was improved. Compared with the traditional inner-cone bond-type anchorage, the radial dimension of this novel composite anchorage only increased the thickness of the clamp. However, the length of the anchorage decreased, largely because of a relative sufficient confinement force by the mechanical part under pre-tightening force and pullout force.

**Figure 2:**
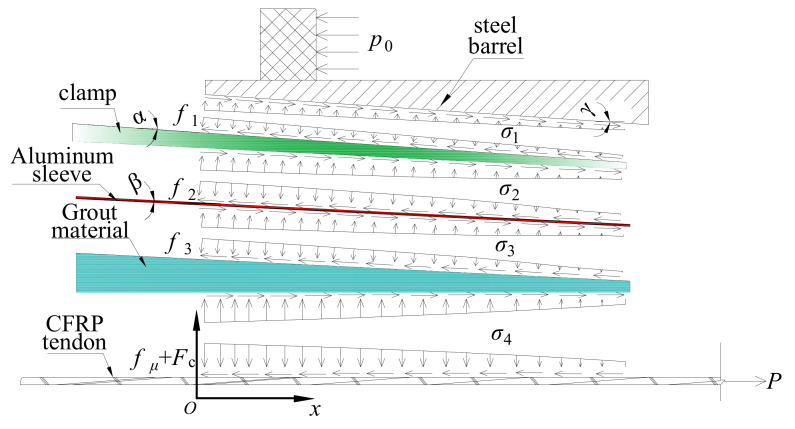
Force analysis of the novel composite anchorage.

Figure 2 depicts the force analysis of each part of the novel anchorage under pullout load, *P*. Only half of this anchorage is shown here, as this anchorage is a central symmetric structure. The load-carrying capacity of this anchorage, $F_{pu}$, can be expressed as follows:

(1) $$F_{pu}=f_{\mu }+F_{c}=\int_{0}^{l}\mu \sigma_{4}(x)C_{f}dx+F_{c}$$

where $f_{\mu }$ is the friction force between the CFRP tendon and the grout material, $F_{c}$ represents the chemical adhesion between the CFRP tendon and the grout material, μ is the friction coefficient between the CFRP tendon and the grout material, $\sigma_{4}(x)$ denotes the contact pressure along the tendon–grout surface, $C_{f}$ is the circumference of the CFRP tendon, and l is distribution length of contact pressure.

For the clamps, the following relationship can be obtained:

(2) $$\int_{0}^{l}\sigma_{1}(x)C_{1}(x)\cos\alpha dx=\frac{\cos\beta -\mu_{2}\sin\beta }{\cos\alpha -\mu_{1}\sin\alpha }\int_{0}^{l}\sigma_{2}(x)C_{2}(x)\cos\beta dx$$

where $\sigma_{1}(x)$ and $\sigma_{2}(x)$ are the contact pressures along the barrel-clamp and clamp-sleeve interfaces, respectively; $C_{1}(x)$ and $C_{2}(x)$ are the circumferences of the outer and inner surfaces of the clamp, respectively; $\mu_{1}$ and $\mu_{2}$ are the friction coefficients along the barrel–clamp and clamp–sleeve interfaces, respectively; and α and β are the inclination angles of the outer and inner surfaces of the clamp, respectively.

For the aluminum sleeve, Equation (3) can be deduced according to Figure 2:

(3) $$\int_{0}^{l}\sigma_{2}(x)C_{2}(x)dx=\frac{1-\mu_{3}\sin\beta }{1-\mu_{2}\sin\beta }\int_{0}^{l}\sigma_{3}(x)C_{3}(x)dx$$

where $\mu_{3}$ refers to the friction coefficient along the sleeve–grout interface, $\sigma_{3}(x)$ is the contact pressure along the sleeve–grout interface, and $C_{3}$ is the circumference of the inner surface of the sleeve. By analyzing the grout material, the contact pressures along the sleeve–grout interface, $\sigma_{3}(x)$, and the grout–tendon interface, $\sigma_{4}(x)$, can be expressed as:

(4) $$(1-\mu_{3}\sin\beta )\int_{0}^{l}\sigma_{3}(x)C_{3}(x)\cos\beta dx=\int_{0}^{l}\sigma_{4}(x)C_{f}dx$$

After the anchorage was assembled, a pre-tightening force, *P*_1_, was exerted on the free ends of the clamps to realize active confinement on the bond part by the mechanical part. Hence, the distribution length of contact pressure, l, in Equations (1)–(4) is the advance volume of the clamp under the pre-tightening force. l can be solved by the radial deformations of each part of the anchorage, Δ, as follows:

(5) $$l=\frac{\Delta }{\tan\alpha }\approx \frac{\Delta }{\alpha }$$

where $\Delta =\Delta_{1}+\Delta_{2}+\Delta_{3}+\Delta_{4}+\Delta_{5}$, $\Delta_{1}∼\Delta_{5}$ represent the radial deformations of the barrel, the clamps, the sleeve, the grout material, and the CFRP tendon, respectively. 

Equations (1)–(5) show that as the contact stress and its distribution length increase, the load-carrying capacity also increases. The radial deformation of each part is closely related to the elastic modulus. A low elastic modulus can lead to a large radial deformation, Δ, and a long distribution length of the anchorage; thus, a thin, soft aluminum sleeve instead of steel was designed as the outer holder of the grout material. On the one hand, the relatively low elastic modulus increased the distribution length of the contact pressure, leading to a more evenly distributed contact pressure and an ideal anchoring performance. On the other hand, when the dimensions of grouted material are determined, a thin, aluminum sleeve can decrease the volumes of the clamps and the outer steel barrel, enabling a high contact pressure under the pre-tightening force. Then, the distribution length of the contact pressure is further improved. Moreover, the soft aluminum sleeve has low hardness, and the clamps can easily grip the bond part by nipping into the aluminum.

To sum up, from the design concept perspective, the novel composite presented in this study has the following advantages over current anchorages: (1) Compared with traditional inner-cone bond-type anchorages, the radial dimensions of this novel composite anchorage only increase the thickness of the clamp (less than 1 cm). However, the pre-tightening force exerted on the clamps can provide a profound active confinement effect on the bond part and can largely decrease the embedded length of the anchorage. (2) The existence of the inclination differential between the outer surface of the clamps and the inner surface of the steel barrel could avoid the stress concentration phenomenon at the loaded end. (3) The thin, soft aluminum sleeve could largely improve the distribution length of the contact pressure and promote anchoring performance.

## 3. Experimental Verification of the Novel Composite Anchorage

### 3.1. Preparation of the Experiments

**Figure 3:**
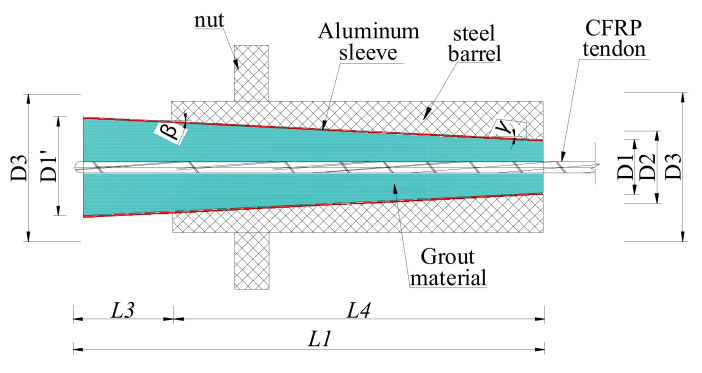
Scheme of the inner-cone bond-type anchorage.

A composite anchorage and an inner-cone bond-type anchorage were fabricated and tested. Their anchoring performances were compared to verify the novelty of this composite anchorage. Figure 3 shows the scheme of the inner-cone bond-type anchorage. Compared with the novel composite anchorage, the inner-cone bond-type anchorage had no clamp. The grouted material was cast in the aluminum sleeve and was cured for 7 days to obtain its final mechanical properties. Then, the grout and the aluminum sleeve were assembled with the outer steel barrel. Table 1 shows the detailed dimensions of the composite anchorage and the inner-cone bond-type anchorage. The dimensions of the bond parts of the composite anchorage and the inner-cone bond-type anchorage were the same. The composite anchorage was named “CER-1A (B)”, whereas the inner-cone bond-type anchorage was named “ICER-1A (B)”. A and B represent the A and B end of the anchorage, respectively.

**Figure 4:**
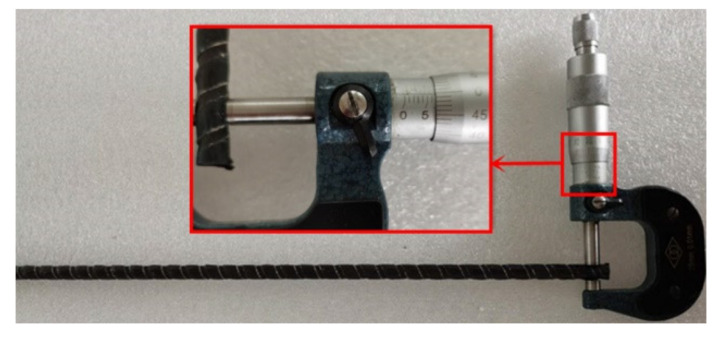
Layout of the CFRP tendon.

For the steel barrel and the clamps, we adopted high-quality steel with a carbon content of 0.45%; the mechanical properties of the steel are shown in Table 2 with reference to the Chinese code for quality carbon structural steels (GB/T 699-2015) [38]. The aluminum sleeve was made from a 1 mm thick aluminum plate, in which the aluminum content reached 99.6% and the mechanical properties of which are also listed in Table 2 according to Chinese code for wrought aluminum and aluminum alloy plates, sheets, and strips for general engineering (Part 2: Mechanical properties; GB/T 3880.2-2012) [39]. For the grouted material, we adopted a commercial epoxy resin Lica, which was made by mixing components A and B with a proportion of 1:2. The mechanical properties of the epoxy resin Lica were provided by the manufacturer and are summarized in Table 3. As shown in Figure 4, a ribbed CFRP tendon with an immersion diameter, *d*, of 7 mm and a nominal cross-sectional area of 34.21 mm^2^ was adopted. The mechanical properties of the CFRP tendon were provided by the manufacture and are listed in Table 4. The linear elasticity and the elastic modulus of the CFRP tendon were verified in our previous study [18], showing the reliability of the CFRP tendon data provided by the manufacturer. 

**Figure 5:**
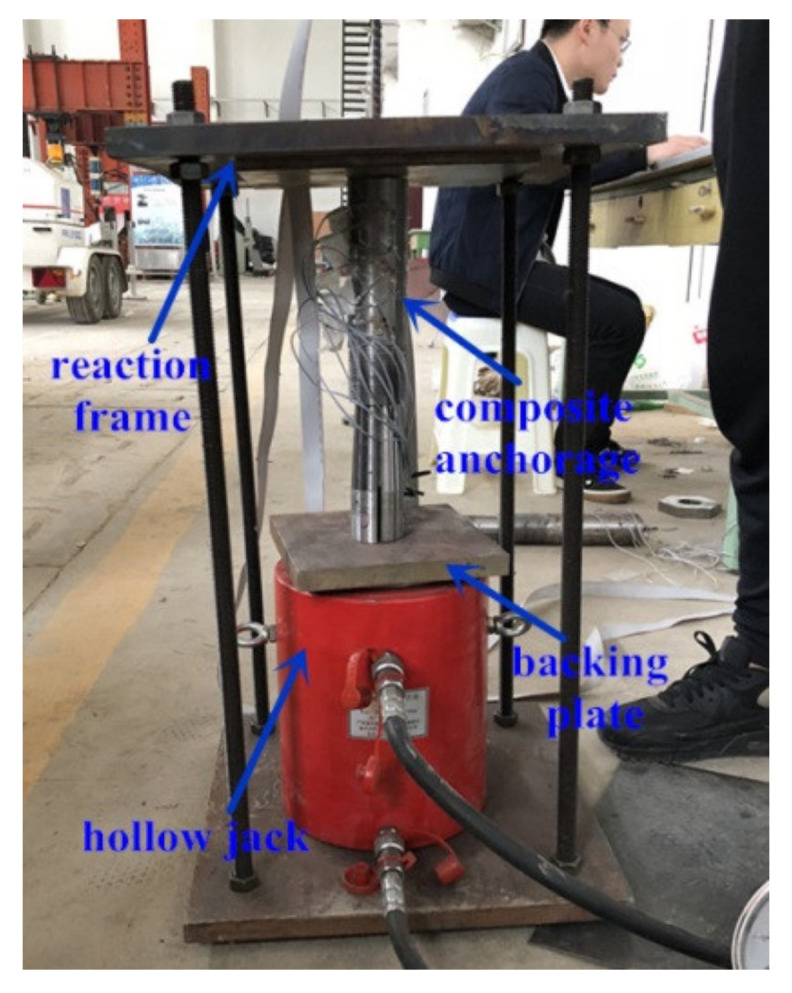
Pre-tightening equipment for the composite anchorage.

**Figure 6:**
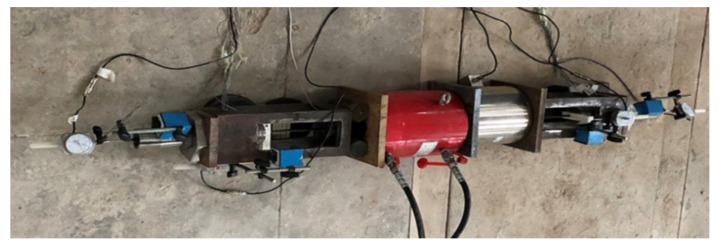
Pullout test device for the composite anchorage.

A pre-tightening procedure was conducted before the anchorage was subjected to the pullout test to exert active confinement on the bond part. As the inner-cone bond-type anchorage had no clamp, a pre-tensioning procedure was conducted on the inner-cone bond-type anchorage by pulling the anchorage beforehand. The pre-tightening and pre-tensioning forces were determined by controlling the front ends of the clamps, grout material, and the steel barrel to be in the same plane as soon as possible. Through this principle, the pre-tightening forces for anchorages CER-1A and CER-1B were 112 and 135 kN, respectively. The pre-tensioning force for anchorage ICER-1 was 32 kN. Figure 5 and Figure 6 show the pre-tightening and pullout test devices, respectively.

**Figure 7:**
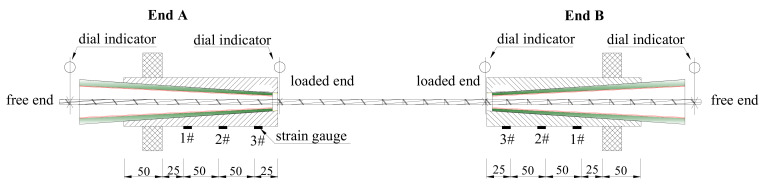
Test apparatus and arrangement of measuring points (unit: mm).

The load–slip relationship is one of the most important indices for anchorages with CFRP tendons. In this test, a hollow pressure transducer was adopted to record the pullout load exerted by the hollow jack. Dial indicators were installed at the free and loaded ends to monitor the slip of CFRP tendons under each load level, as shown in Figure 7. Strain gauges were also attached on the surface of the steel barrel at equal spacing to record the hoop stress characteristics of the anchorage.

### 3.2. Test Results

**Figure 8:**
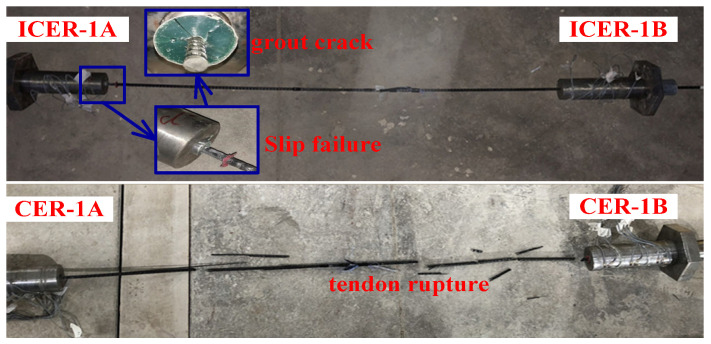
Failure modes of anchorages CER-1 and ICER-1.

**Figure 9:**
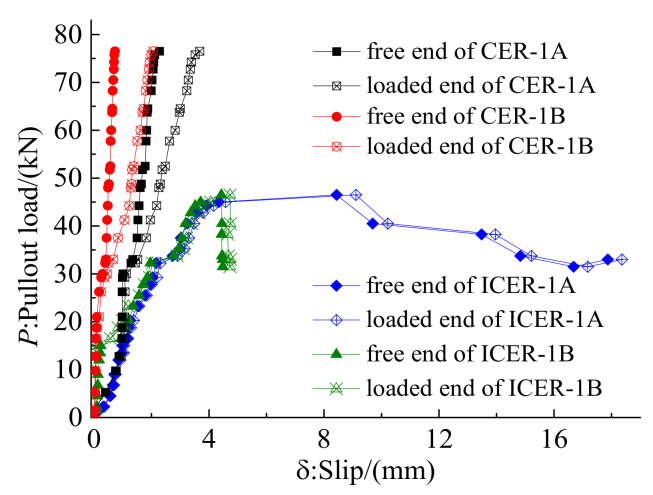
Test load–slip relationship for anchorages CER-1 and ICER-1.

Figure 8 depicts the failure modes of anchorages CER-1 and ICER-1. The composite anchorage, CER-1, failed by tendon rupture in the free zone of the anchorage, whereas the inner-cone bond-type anchorage, ICER-1, exhibited a tendon-slip failure. Figure 9 shows the load–slip curves of anchorages CER-1 and ICER-1. As shown in Figure 9, the slips at the free and loaded ends of anchorage CER-1 were evidently smaller than those of anchorage ICER-1. In addition, the load-carrying capacity of anchorage CER-1 was 76.49 kN, which is much larger than that of anchorage ICER-1 (46.50 kN). 

In addition, the Chinese code for anchorage, grip, and couplers for prestressing tendons (GB/T 14370-2015) [40] specifies the anchoring efficiency, $\eta_{a}$, for pre-stressed anchorage as follows:

(6) $$\eta_{a}=\frac{F_{npu}}{\eta_{p}F_{pm}}$$

where $F_{npu}$ represents the load-carrying capacity of the anchorage; $\eta_{p}$ is the reduction efficiency of the pre-stressed tendons and is related with the number of the pre-stressed tendons, *n*, when *n* = 1~5, $\eta_{p}=1.0$; when *n* = 6~12, $\eta_{p}=0.99$; when *n* = 13~19, $\eta_{p}=0.98$; when *n* ≥ 20, $\eta_{p}=0.97$. $F_{pm}$ denotes the theoretical tensile-bearing capacity of the pre-stress tendons and can be expressed as Equation (7):

(7) $$F_{pm}=n\times A_{pk}\times f_{ptk}$$

where $A_{pk}$ and $f_{ptk}$ are the cross-sectional area and tensile strength of the pre-stressed tendons, respectively.

The tensile-bearing capacity of the CFRP tendon was calculated as 78.68 kN by Equation (7) based on its mechanical properties. Then, the anchoring efficiency of anchorages CER-1 and ICER-1 was calculated as 0.97 and 0.59, respectively. The anchoring efficiency of anchorage CER-1 was 60.4% higher than that of anchorage ICER-1. However, the diameter of anchorage CER-1 was only 10.5% larger than that of anchorage ICER-1. For anchorage ICER-1, a longer embedded length was needed to successfully grip the CFRP tendon. Hence, the novel composite anchorage was able to successfully grip the CFRP tendon with a slightly larger diameter but with a much shorter embedded length compared with traditional inner-cone bond-type anchorages. Moreover, the failure mode and the anchoring efficiency satisfied the provisions that stipulated that the failure mode for an FRP tendon anchorage should be tendon rupture instead of slip failure and the anchoring efficiency of an FRP tendon anchorage should not be less than 0.9 (GB/T 14370-2015) [40]. The failure mode, slip value, and load-carrying capacity (anchoring efficiency) proved that the novel composite anchorage had a superior anchorage performance compared to the traditional inner-cone bond-type anchorage.

**Figure 10:**
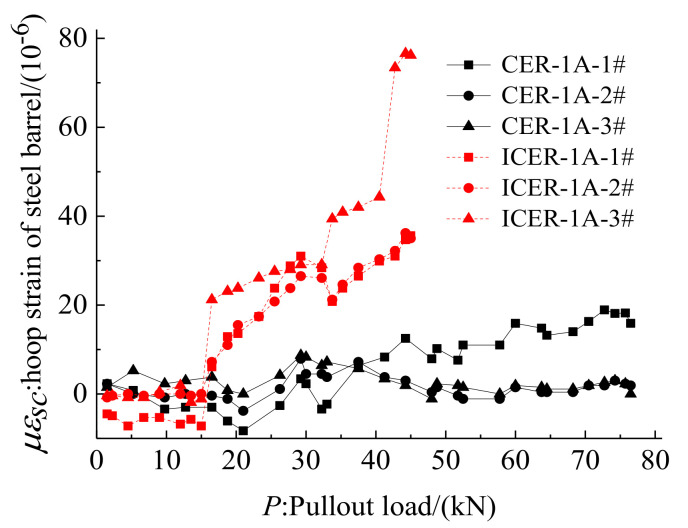
Hoop strain of steel barrel for anchorages CER-1 and ICER-1.

Figure 10 also shows the hoop strain distribution of the outer steel barrel for anchorages CER-1 and ICER-1. As shown in Figure 10, the peak strain of anchorage CER-1 occurred near the free end of the anchorage. However, the peak strain of anchorage ICER-1 concentrated near the loaded end of the anchorage. Furthermore, the peak strain of anchorage CER-1 was lower than that of anchorage ICER-1. This finding proved that the novel composite anchorage could effectively address the stress concentration problem at the loaded end of the inner-cone anchorage and could improve the anchoring performance by immigrating the peak stress from the loaded end to the free end. In addition, although the peak stress was transferred from the loaded end to the free end, the peak stress was relatively low. In other words, no evident vulnerable position was found at the junction of the mechanical and bond parts as with traditional composite anchorage, as reported in [32]. Thus, the advantage of this novel composite anchorage over traditional composite anchorages in avoiding the occurrence of vulnerable positions was further verified.

## 4. Parametric Study

Once an anchorage is designed, its optimal design details need to be proposed for convenient application. As the proposed composite anchorage is composed of different parts and needed to be pre-tightened before it was assembled, the design details and the pre-tightening force influenced the mechanical properties of the anchorage. In this paper, the influences of the pre-tightening force, *P*_1_; the inclination angle of the bond part β; the inclination differential, (α−γ); and the embedded length, *L*_1_, on the anchoring performance were studied using the FE method.

### 4.1. FE Model

**Figure 11:**
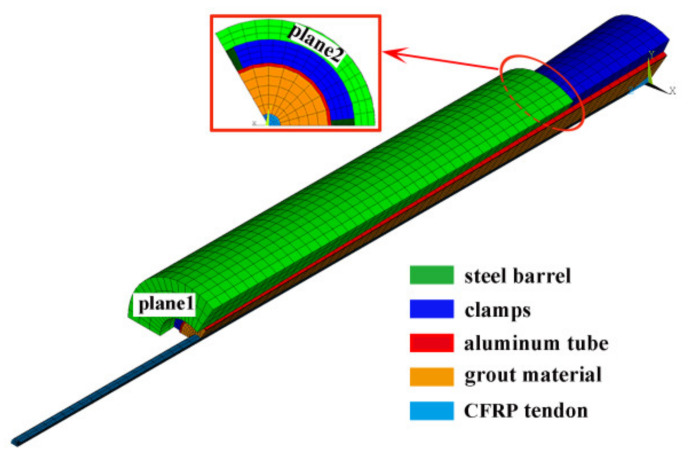
FE model of the composite anchorage.

Three consecutive procedures were conducted to establish an FE model of this novel composite anchorage. First, a solid model containing each part was built. Then, interfacial behaviors between each part were modeled. Finally, the boundary conditions and load cases were simulated. The FE model was built and simulated using ANSYS 19 software (Canonsburg, PA, USA). When modeling the solid structure of each part, the SOLID 65 element was adopted. The steel barrel, clamps, and the aluminum sleeve were modeled as isotropic linear elastic materials, whereas the grout material and the CFRP tendon were modeled as anisotropic linear materials. The material properties of each part were taken from Table 2, Table 3 and Table 4. Only one-third of this anchorage was modeled for computing convenience, as this anchorage is a central, symmetric structure. Figure 11 shows the FE model.

Interfacial behavior was simulated using a surface–surface contact element, in which the contact surface was modeled with the CONTA 173 element and the target surface adopted the TARGE 170 element. The contact stiffness of the contact surface between the steel barrel and the clamps was 0.35, whereas the contact stiffnesses of the contact surfaces between the clamps and aluminum sleeve and between the grouted materials and the CFRP tendon were 0.25. The clamps exerted a high contact pressure on the surface between the aluminum sleeve and the grouted material, and slip hardly occurred along the sleeve–grout interface. Thus, the aluminum sleeve and grout material were considered well bonded and unseparated in the FE model. The contact algorithm adopted a combination of Lagrange multiplier and penalty function methods.

In addition, a pre-tightening procedure was designed before the anchorage was subjected to pullout load, so different load cases and boundary conditions were considered in different procedures in this model. To completely simulate the pre-tightening and pullout procedures of this anchorage, the following four load steps were involved:

(1) Exerting the pre-tightening force. As mentioned above, a pre-tightening procedure was designed to produce active confinement; the first step was to simulate the pre-tightening behavior. To achieve this, the front plane of the steel barrel (plane 1) was constrained, and pre-tightening force was exerted on the free end plane of the clamp in terms of uniform distributed load.
(2) Removing the pre-tightening force. In this step, the pre-tightening force was removed from the clamp. The confining effect of the pre-tightening force still existed, as the boundary condition remained unchanged.
(3) Converting the boundary condition. The boundary condition in Step (1) did not agree with the real case in the pullout procedure; thus, the boundary condition needed to be changed. In this step, the constraint of plane 1 was removed, and the plane 2 (free-end plane of steel barrel) was constrained at the same time. In this step, no actual load was exerted, but the boundary condition was converted.
(4) Pulling the anchorage. In this final step, a gradually increased pullout load was exerted on the front plane of the CFRP tendon from 0 to the failure of the anchorage to accomplish the pullout procedure.

**Figure 12:**
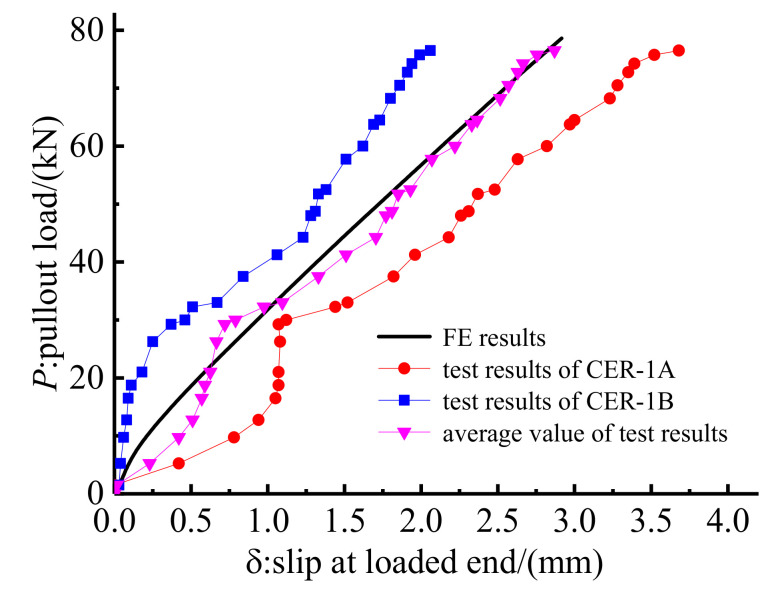
Comparison between test and FE results of anchorage CER-1.

After the FE model was simulated, the validity of the FE model was proven by comparing the FE and test results for anchorage CER-1. Figure 12 shows the load–slip relationship at the loaded end of anchorage CER-1 obtained using FE and test methods. As shown in Figure 12, the load–slip curves of anchorages CER-1A and CER-1B deviate from one another, although they were designed with the same parameters. The slip of anchorage CER-1A is larger than that of anchorage CER-1B. Compared with anchorage CER-1B, the slip of anchorage CER-1A increases rapidly when the pullout load is less than 13 kN. Then, the slip begins to slow down with the increase in the pullout load. This is because the pre-tightening force of anchorage CER-1A is less than that of anchorage CER-1B according to the pre-tightening principle. When the anchorage is subjected to pullout load, anchorage CER-1A first compensates the pullout to the difference in pre-tightening force. The pre-tightening force in the FE model adopted the average pre-tightening force of anchorages CER-1A and CER-1B, so the load–slip relationship calculated through FE methods could not match well with the individual test results. Figure 12 also plots the average load–slip relationship for anchorage CER-1. Although the FE results do match well with the individual test results, such results can predict the average load–slip relationship with good agreement. Thus, the FE model is valid to simulate the mechanical properties of the proposed composite anchorage.

### 4.2. Influence of the Pre-Tightening Force, P_1_

After the FE model was validated, a wide range of design parameters was considered by the FE model to determine their influence on the mechanical properties of the proposed composite anchorage. For this composite anchorage, the load–slip relationship and the stress distribution in the anchorage zone were the main indices to evaluate the anchorage performance, so the load–slip relationship and stress distribution were obtained from the FE model for each parameter.

**Figure 13:**
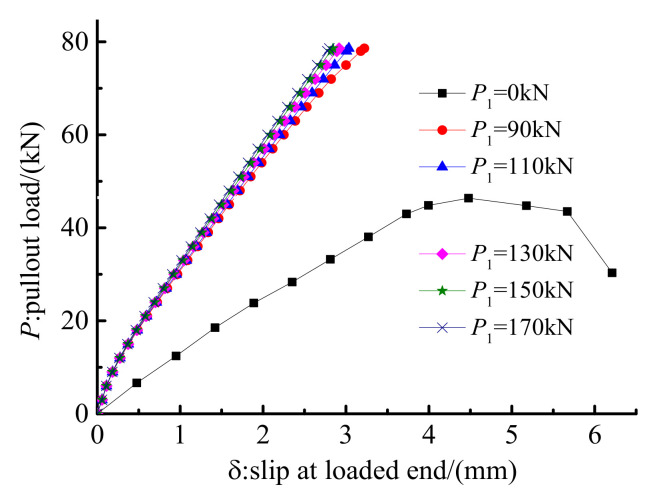
Load–slip relationship of anchorage with different pre-tightening forces.

**Figure 14:**
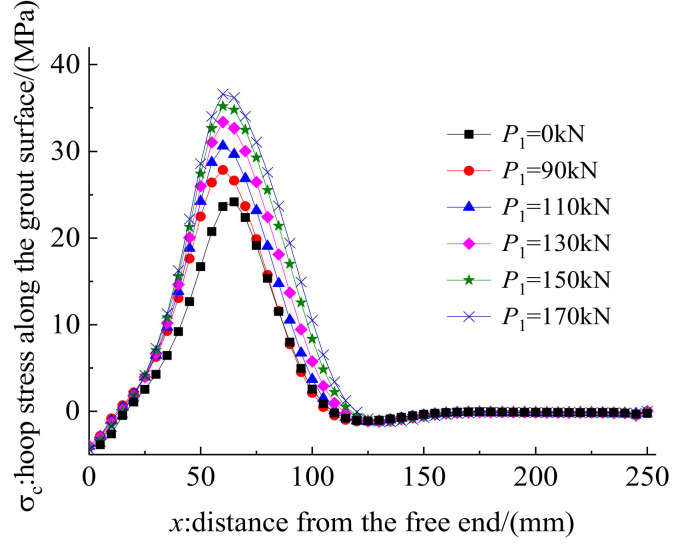
Hoop stress along the grout surface of anchorage with different pre-tightening forces.

Pre-tightening forces of 0, 90, 110, 130, 150, and 170 kN were exerted on the clamps of anchorage CER-1 in the FE model to examine the influence of the pre-tightening force, *P*_1_, on the anchorage performance. Other design parameters remained unchanged when studying the influence of the pre-tightening force. Figure 13 and Figure 14 plot the load–slip relationship and hoop stress on the outer surface of the grout material for different pre-tightening forces. As shown in Figure 13, the pre-tightening force has a remarkable influence on the load–slip relationship. When the pre-tightening force is 0 kN, the anchorage fails by tendon slip, and the slip of the tendon is much larger than the cases when *P *_1_= 90, 110, 130, 150, or 170 kN. However, when pre-tightening forces are 90, 110, 130, 150, and 170 kN, the load-carrying capacities of the anchorages reach the tendon rupture load. When pre-tightening force increases from 90 kN to 110, 130, 150, and 170 kN, the decrease in the tendon slip is not obvious. When the pre-tightening force continues to increase to 110, 130, 150, and 170 kN from 90 kN, the slip only decreases by 5.79%, 3.92%, 2.51%, and 1.53%, respectively. When the pre-tightening force is above 130 kN, the reduction effect on the slip value is limited. This finding shows that although the pre-tightening force has a considerable influence on reducing the slip of the tendon, the extent of the influence decreases with the increase in the pre-tightening force. In other words, reducing the slip by blindly increasing the pre-tightening force is not always effective and is not advisable.

The hoop stress distribution along the outer surface of the grout material reflects the active confinement effect of the mechanical part. Figure 14 shows that the contact pressure is mainly distributed in the zone of 0–125 mm from the free end. This is because the existence of the inclination differential results in a gap between the clamps and the steel barrel near the loaded end. This gap could not be eliminated under pre-tightening and pullout loads. Thus, the contact pressure was mainly distributed near the free end, and the stress concentration phenomenon at the loaded end was avoided. Moreover, Figure 14 shows that the peak hoop stress and the stress distribution length increase as the pre-tightening force increases. The peak hoop stress when pre-tightening force is 0 kN is slightly less than that when the pre-tightening force is 90 kN. This is because the hoop stress when the pre-tightening force is 0 kN is mainly caused by the slips of the clamps and the bond part under pullout load. The hoop stress when the pre-tightening force is 0 kN sacrifices a large slip value of the anchorage and is a passive confinement stress. However, the hoop stress for the anchorage with the pre-tightening force mainly comes from the deformation of each part under the pre-tightening force. The slip caused by the pre-tightening force does not influence the total slip in service, as the pre-tightening procedure is in advance of the pullout procedure. In addition, the peak hoop stresses on the outer surface of the grout under the pullout load when the pre-tightening forces are 90, 110, 130, 150, and 170 kN are 27.9, 30.6, 33.4, 35.2, and 36.6 MPa, respectively. The influence of the pre-tightening force on the confinement effect decreases gradually with the increase in the pre-tightening force. When the pre-tightening force exceeds 130 kN, the promotion effect of the pre-tightening force on the hoop stress is not significant. A pre-tightening force of 130 kN is advised for the proposed composite anchorage by comprehensively considering the influence of pre-tightening force on the slip and hoop stress values.

### 4.3. Influence of the Inclination Differential (α−γ)

**Figure 15:**
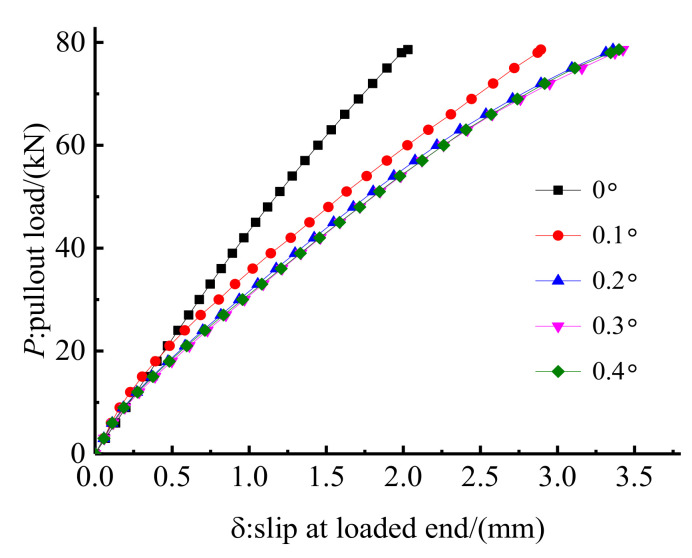
Load–slip relationship of anchorage with different inclination differentials.

Five inclination differentials of 0°, 0.1°, 0.2°, 0.3°, and 0.4° were simulated through FE methods to study the influence of the inclination differential on the anchorage performance. Other design parameters of anchorage CER-1 remained unchanged. Then, the load–slip relationship and hoop stress distribution under pullout load with a pre-tightening force of 90 kN were calculated. Figure 15 depicts the load–slip relationships of the anchorage with different inclination differentials. The anchorage without inclination differential exhibits the least slip, followed by that with an inclination differential of 0.1°. In contrast, the slips of anchorages with inclination differentials of 0.2°, 0.3°, and 0.4° are nearly the same and are slightly larger than that with an inclination differential of 0.1°. This result implies that the influence of inclination differential on the anchoring performance is nonlinear.

**Figure 16:**
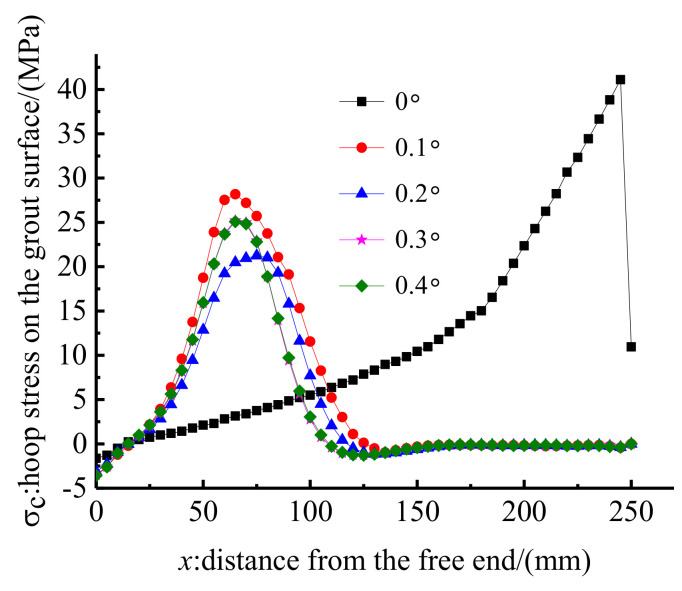
Hoop stress along the grout surface of anchorage with different inclination differentials.

Figure 16 shows the hoop stress along the outer surface of the grout material. The stress distribution features of the anchorage without inclination differential is different from those of anchorages with inclination differential of 0.1°, 0.2°, 0.3°, and 0.4°. For anchorage without an inclination differential, the peak hoop stress occurs at the loaded end of the anchorage, whereas the peak stress of anchorages with inclination differentials occurs near the free end of the anchorage. In essence, the anchorage without inclination differential exhibits a similar anchoring performance to that of the traditional inner-cone bond-type anchorage, except for the existence of the active confinement effect of the pre-tightening force. This notion also proves the rationality of setting an inclination differential to avoid the stress concentration phenomenon for the proposed composite anchorage. In addition, the enveloping area of the hoop stress curve represents the total confinement effect of the clamps on the bond part. As shown in Figure 16, the hoop stress and its distribution zone of anchorage with an inclination differential of 0.1° are larger than those of anchorages with inclination differentials of 0.2°, 0.3°, and 0.4°. The confinement effect of the anchorage with inclination differential of 0.1° is more profound, and the slip is less than that of anchorages with inclination differentials of 0.2°, 0.3°, and 0.4°. The stress distribution curves of anchorages with inclination differentials of 0.3° and 0.4° almost coincide; thus, the confinement effects and load–slip relationships of these two anchorages are nearly the same. The hoop stress of the anchorage with an inclination differential of 0.2° is smaller than that of anchorages with inclination differential of 0.3° and 0.4°. However, the stress distribution zone of the anchorage with an inclination differential of 0.2° is larger than that of anchorages with inclination differentials of 0.3° and 0.4°. The envelope areas of the hoop stress curves of these three anchorages are nearly the same; hence, the load–slip relationships of these three anchorages show a limited difference. To avoid the stress concentration phenomenon at the loaded end and to meet a small slip, an inclination differential of 0.1° is proposed in designing this composite anchorage.

### 4.4. Influence of the Inclination Angle, β

**Figure 17:**
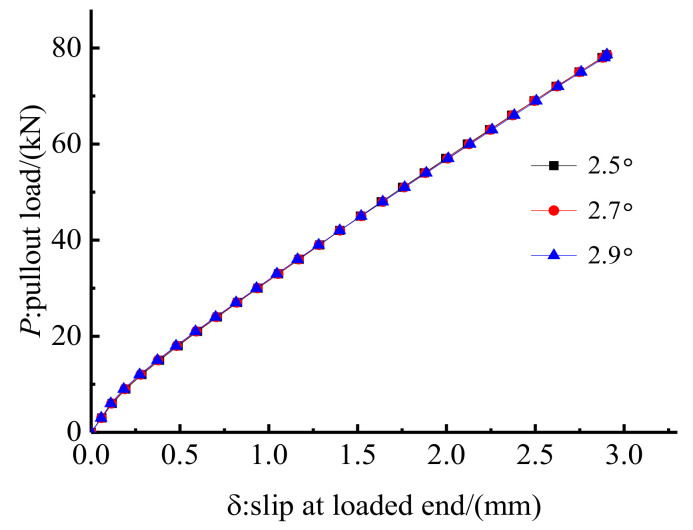
Load–slip relationship of anchorage with different inclination angles.

**Figure 18:**
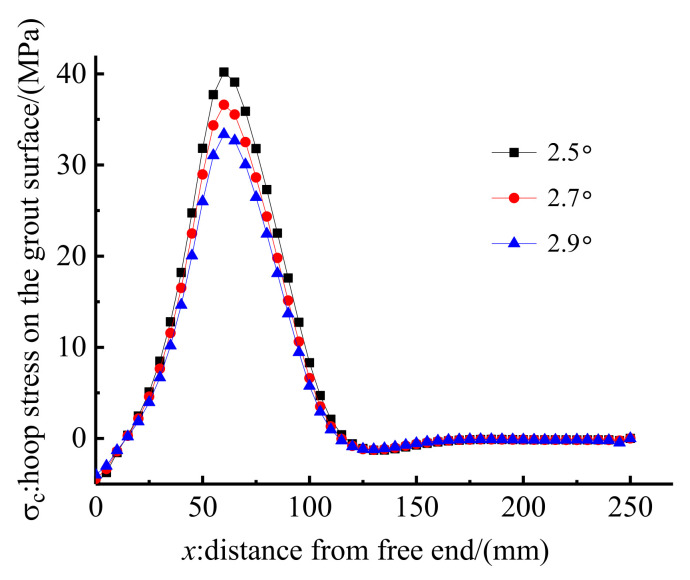
Hoop stress along the grout surface of with different inclination angles.

Three inclination angles of 2.5°, 2.7°, and 2.9° were selected to study the influence of inclination angle on the anchoring performance of the proposed composite anchorage. Other designed parameters were kept the same for anchorage CER-1 in the test. Figure 17 shows the load–slip relationships of anchorages with different inclination angles. As shown in Figure 17, the inclination angle has a limited influence on the load–slip relationship, and the load–slip relationships of anchorages with different inclination angles show little difference. Figure 18 shows the hoop stress distribution. As shown in Figure 18, the stress distribution zones of anchorages with different inclination angles are nearly the same, but the peak stress differs. The peak stress for anchorages with inclination angles of 2.5°, 2.7°, and 2.9° are 40.2, 36.6, and 33.4 MPa, respectively. This is because the volume of the grout material decreases correspondingly as the inclination angle decreases. The stress of grout material with a lower volume is higher than that of grout material with a higher volume under the same pre-tightening force. When the slips of different anchorages are at the same level, an anchorage with low hoop stress is advised, as high hoop stress may result in the crushing of the grout material. Moreover, when the inclination angle is small, a small pre-tightening force can result in the front ends of the clamps and the steel barrel on the same plane in practice [18]. In other words, the confinement effect of anchorages with a small inclination angle is insufficient in practice. Hence, a large inclination angle is needed. However, a large inclination angle results in large anchorage dimensions, which will influence the applicability of the anchorage. An inclination angle of 2.9° is recommended for this composite anchorage, considering an ideal confinement effect that is also unapt to crush the grout material through FE and experimental studies.

### 4.5. Influence of the Embedded Length, L_1_

**Figure 19:**
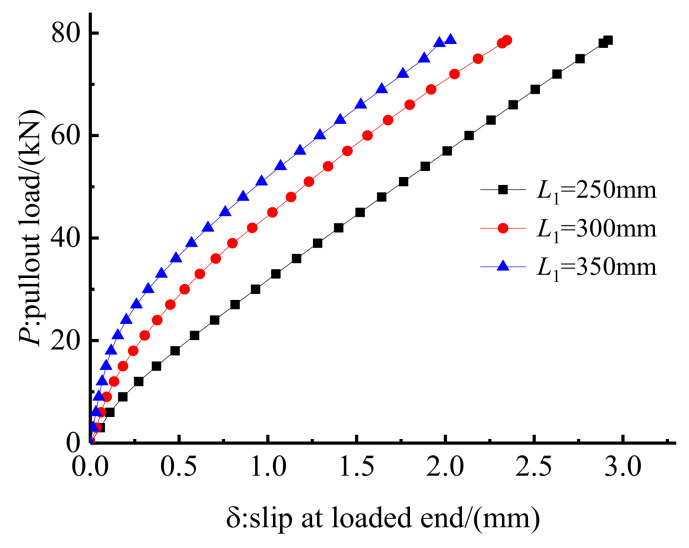
Load–slip relationship of anchorage with different embedded lengths.

**Figure 20:**
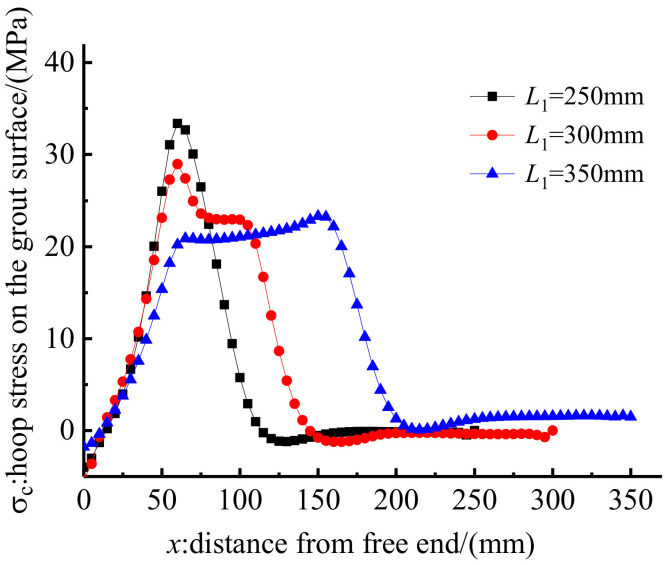
Hoop stress along the grout surface of anchorage with different embedded lengths.

The influence of the embedded length on anchorage performance was also investigated through the FE method by changing the embedded length of anchorage CER-1 from 250 mm to 300 and 350 mm. Figure 19 and Figure 20 show the load–slip relationship and the hoop stress distribution of anchorages with different embedded lengths. Figure 19 shows that if the embedded length is long, then the slope of the load–slip curve is steep and the slip of the anchorage is small. The hoop stress distribution in Figure 20 can partially explain the internal reason. As shown in Figure 20, although the peak hoop stress decreases with the increase in the embedded length, the distribution length of hoop stress increases with the embedded length. The enveloping area of the hoop stress distribution curve also increases with the embedded length. The confinement effect of the anchorage with a longer embedded length is also larger than that of anchorages with shorter embedded lengths. Furthermore, with the increase in the embedded length, the chemical adhesion between the grout and the CFRP tendon also increases correspondingly. Thus, the internal reason why a long embedded anchorage exhibits a good load–slip relationship is explained.

For the proposed composite anchorage, as the embedded length increases, the radial dimensions also increase. In engineering practice, extremely large dimensions of the anchorage will affect its applicability. In the test, an embedded length of 250 mm was sufficient to grip the CFRP tendon. Based on the establishment of a linkage between the embedded length and the diameter of the CFRP tendon, an embedded length of 30 *d*~40 *d* is proposed for the design of this composite anchorage.

## 5. Performance of the Composite Anchorage for Multiple CFRP Tendons

**Figure 21:**
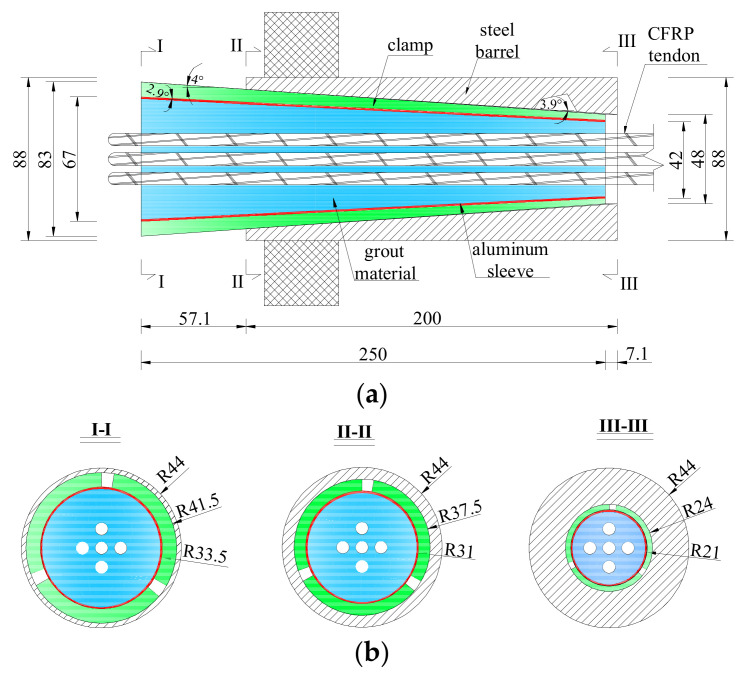
Scheme of anchorage CER-5. (unit: mm) (**a**) longitudinal section; (**b**) cross section.

A composite anchorage with five CFRP tendons, labeled as CER-5, was designed and tested to verify the reliability of the proposed composite anchorage for gripping multiple CFRP tendons. The design parameters were selected according to a parametric study and the design proposals in Section 4, listed as follows: the inclination angle of the grout, β, is 2.9°; the inclination angles of the steel barrel, γ, and the outer surface of the clamp, α, are 3.9° and 4°, respectively. The inclination differential is 0.1°; consequently, the embedded length is 250 mm, and the pre-tightening force is 130 kN. The clear space between the central and outer CFRP tendons was set at 7 mm. Figure 21 shows a cross-sectional diagram of the proposed anchorage. The test procedure for anchorage CER-5 was the same as that for anchorage CER-1.

**Figure 22:**
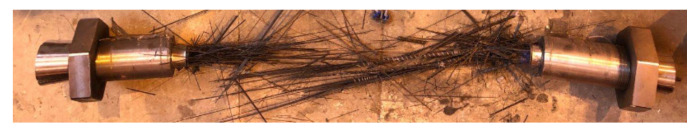
Failure mode of anchorage CER-5.

As shown in Figure 22, anchorage CER-5 failed with the rupture of CFRP tendons when the pullout load reached 413.20 kN. The fibers of the tendons scattered when the anchorage failed. The failure mode of anchorage CER-5 satisfied the provision that stipulates that the failure mode for FRP tendon anchorage should be tendon rupture instead of slip failure (GB/T 14370-2015) [40]. In addition, the anchoring efficiency of anchorage CER-5 was calculated as 1.05 by substituting $F_{npu}=413.2\;\mathrm{kN}$ into Equation (6), which also satisfies the provision that the anchoring efficiency of FRP tendon anchorage should not be less than 0.9 (GB/T 14370-2015) [40]. The ideal failure mode and anchoring efficiency proved that the design parameters suggested in Section 4 were reliable for this composite anchorage to grip multiple CFRP tendons.

**Figure 23:**
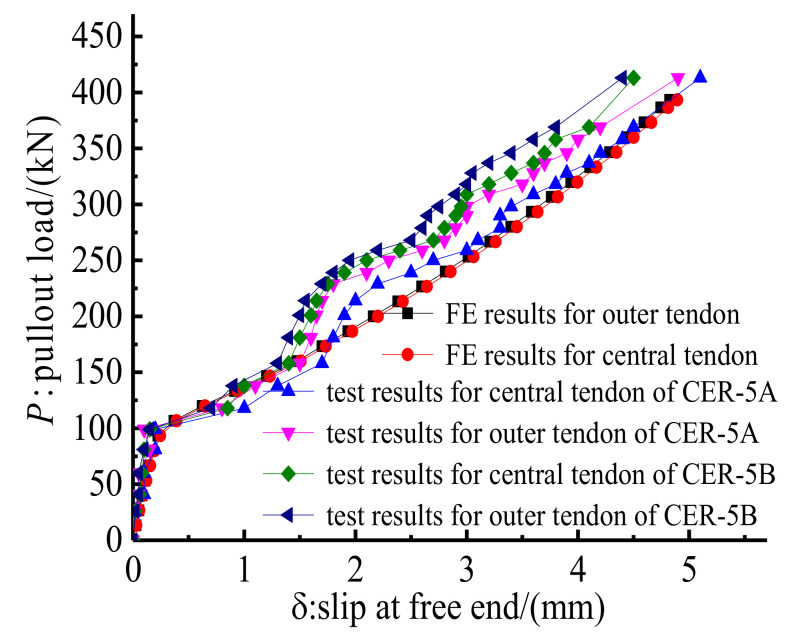
Test and FE load–slip relationships for anchorage CER-5.

**Figure 24:**
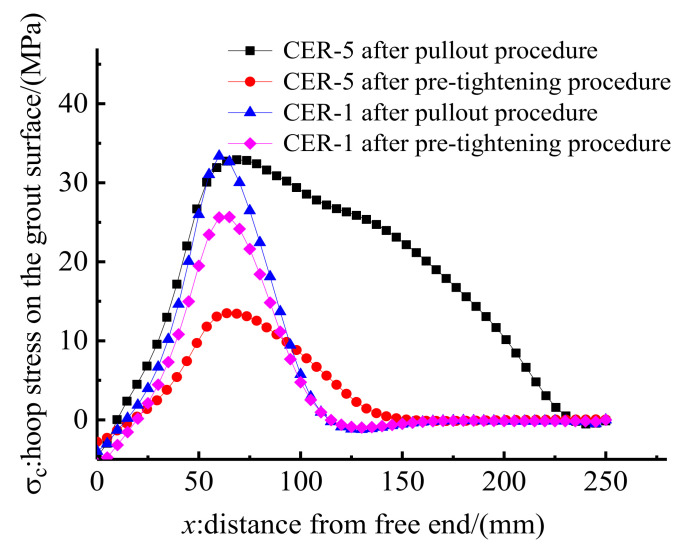
Hoop stress on the grout surface for CER-1 and CER-5.

Anchorage CER-5 contained a central tendon and four outer tendons. Thus, the slips of the central tendon and one of the four outer tendons at the loaded end of anchorages CER-5A and CER-5B were tested to examine the mechanical performance difference of central and outer CFRP tendons. Figure 23 shows the load–slip relationships of the central and outer tendons by test and FE methods. FE results (Figure 23) are in agreement with the test results, which further prove the validity of the FE model. In addition, the test and FE results in Figure 23 show that the slip of the central CFRP tendon is slightly larger than that of the outer CFRP tendon. This finding may be attributed to the non-uniformity in the stress distribution between the central and outer CFRP tendons. The longitudinal deformation of the grout material in the cross section decreases from the central point to the edge of the grout material. Thus, the bond stiffness between the outer CFRP tendon and the grout is larger than that between the central CFRP tendon and the grout. Moreover, the hoop stress distribution along the outer surface of the grout material of anchorage CER-5 by the FE model is also plotted and compared with that of anchorage CER-1 in Figure 24. The hoop stress distribution zones for anchorages CER-1 after pre-tightening and pullout procedures are small. However, the hoop stress distribution zones for anchorage CER-5 after pre-tightening and pullout procedures differ considerably. After the pullout procedure, the hoop stress distribution zone for anchorage CER-5 increases to almost occupy the whole length of the anchorage. This is because anchorage CER-5 bears a larger pullout load than anchorage CER-1, and the clamps and bond part of anchorage CER-5 slip considerably more toward the loaded end than anchorage CER-1 under pullout load. The gap between the clamps and the steel barrel of anchorage CER-5 is smaller than that of anchorage CER-1, and the contact area of the clamps and steel barrel of anchorage CER-5 is larger than that of anchorage CER-1. Hence, the passive confinement effect of CER-5 is more evident than that of anchorage CER-1, and the hoop stress of anchorage CER-5 is more uniformly distributed than that of anchorage CER-1. Despite this, the peak stress does not occur at the loaded end but near the free end of the anchorage. This notion also proves the reliability and rationality of this composite anchorage in gripping multiple CFRP tendons.

## 6. Conclusions

A novel composite anchorage for CFRP tendons was proposed in this study. The advantages of this novel composite anchorage were studied through experimental and FE methods. Then, the optimal design parameters were recommended by a parametric study, and the composite anchorage with multiple CFRP tendons with the suggested design parameters was tested to validate the rationality of the optimal design parameters. Based on our study, we propose the following conclusions:

(1) Compared with the traditional inner-cone bond-type anchorage, the novel composite anchorage proposed in this study not only increased the load-carrying capacity by 60.4% and reduced the slip of the CFRP tendon but also avoided the stress concentration phenomenon at the loaded end of the anchorage. The failure mode of the novel anchorage was tendon rupture, and the anchoring efficiency satisfied the related provisions. In addition, compared with traditional composite anchorages, the proposed novel composite anchorage had smaller dimensions and avoided the presence of vulnerable positions at the junction of the mechanical and bond parts.
(2) The FE model simulated mechanical properties of this novel composite anchorage with good agreement. The FE results showed that the pre-tightening force had a considerable influence on the mechanical properties of the proposed composite anchorage. However, the influence of the pre-tightening force on the mechanical properties decreased with the increase in pre-tightening force. Based on a comprehensive consideration of the influence of pre-tightening force on the slip and hoop stress values, a pre-tightening force of 130 kN was suggested.
(3) The existence of an inclination differential of the proposed novel composite anchorage effectively avoided the stress concentration phenomenon at the loaded end. However, the larger the inclination differential was, the smaller the confinement effect was and the larger the slip of the anchorage. An inclination differential of 0.1° was proposed to avoid the stress phenomenon at the loaded end and to promote a good confinement effect.
(4) FE results showed that the inclination angle had a limited influence on the load–slip relationship. Nevertheless, a small inclination angle resulted in high hoop stress, which might crush the grout. Based on consideration of an ideal confinement effect, which was also unapt to crush the grout material through FE and experimental studies, an inclination angle of 2.9° was recommended for the proposed composite anchorage.
(5) A long embedded length means a uniformly distributed contact stress and a small slip of the anchorage. However, the increase in the embedded length also increased the radial dimensions, which would influence the applicability. Considering that the anchorage with an embedded length of 250 mm was sufficient to grip CFRP tendon in the test, an embedded length of 30 d~40 d was advised for different nominal diameters of CFRP tendons.
(6) A composite anchorage with five CFRP tendons was designed with the optimized parameters, and the test results showed that the optimal parameters are rational for this novel composite anchorage to grip multiple CFRP tendons. In addition, the FE and test results showed that the stresses in the central and outer CFRP tendons were not uniform because the bond stiffness between the outer CFRP tendon and the grout was higher than that between the central CFRP tendon and the grout. Moreover, the proposed composite anchorage with multiple CFRP tendons had a better hoop stress distribution than that with a single CFRP tendon, which proved the reliability and rationality of the proposed composite anchorage in gripping multiple CFRP tendons.

## Figures and Tables

**Table 1:** Design details of anchorages CER-1 and ICER-1.

Specimen	*L*_1_ (mm)	*L*_2_ (mm)	*L*_3_ (mm)	*L*_4_ (mm)	*D*_1_ (mm)	*D*_2_ (mm)	*D*_1′_ (mm)	*D*_2′_ (mm)	*D*_3_ (mm)	*α* (°)	*β* (°)	*γ* (°)
CER-1	250	7.1	57.1	200	17	23	42	58	63	4	2.9	3.9
ICER-1	250	/	50	200	17	/	42	/	57	/	2.9	2.9

**Table 2:** Mechanical properties of the steel barrel and aluminum tube.

Material	Elastic Modulus (GPa)	Possion’s Ratio	Ultimate Strength (MPa)
Steel barrel	210	0.31	600
Aluminum sleeve	71	0.31	90

**Table 3:** Mechanical properties of epoxy resin.

Mechanical Properties	Shear Strength (MPa)	Tensile Strength (MPa)	Compressive Strength (MPa)	Elastic of Modulus (GPa)
Results	24.3	40.1	73.6	2.61

**Table 4:** Mechanical properties of CFRP tendon.

Mechanical Properties	Tensile Strength (MPa)	Elastic of Modulus (GPa)	Density (kg/m^3^)	Ultimate Elongation (%)
Results	2300	147	1600	1.44

## Data Availability

The data presented in this study are available on request from the corresponding author.
